# Circular RNA ciRS-7 affects the propagation of *Cryptosporidium parvum* in HCT-8 cells by sponging miR-1270 to activate the NF-κB signaling pathway

**DOI:** 10.1186/s13071-021-04739-w

**Published:** 2021-05-06

**Authors:** Yan-Ling Yin, Ting-Li Liu, Qian Yao, Yu-Xin Wang, Xue-Mei Wu, Xue-Ting Wang, Xin Yang, Jun-Ke Song, Guang-Hui Zhao

**Affiliations:** 1 Department of Parasitology, College of Veterinary Medicine, Northwest A&F University, Yangling, China; 2 State Key Laboratory of Veterinary Etiological Biology, Key Laboratory of Veterinary Parasitology of Gansu Province, Lanzhou Veterinary Research Institute, Chinese Academy of Agricultural Sciences, Lanzhou, China

**Keywords:** *C. parvum*, CircRNAs, CiRS-7/miR-1270/*RelA* axis, Propagation, NF-κB signaling pathway

## Abstract

**Background:**

*Cryptosporidium* is an important zoonotic pathogen responsible for severe enteric diseases in humans and animals. However, the molecular mechanisms underlying host and *Cryptosporidium* interactions are still not clear.

**Methods:**

To study the roles of circRNAs in host cells during *Cryptosporidium* infection, the expression profiles of circRNAs in HCT-8 cells infected with *C. parvum* were investigated using a microarray assay, and the regulatory role of a significantly upregulated circRNA, ciRS-7, was investigated during *C. parvum* infection.

**Results:**

*C. parvum* infection caused notable alterations in the expression profiles of circRNAs in HCT-8 cells, and a total of 178 (including 128 up- and 50 downregulated) circRNAs were significantly differentially expressed following *C. parvum* infection. Among them, ciRS-7 was significantly upregulated and regulated the NF-κB signaling pathway by sponging miR-1270 during *C. parvum* infection. Furthermore, the ciRS-7/miR-1270/*relA* axis markedly affected the propagation of *C. parvum* in HCT-8 cells.

**Conclusions:**

Our results revealed that ciRS-7 would promote *C. parvum* propagation by regulating the miR-1270/*relA* axis and affecting the NF-κB pathway. To the best of our knowledge, this is the first study to investigate the role of circRNA during *Cryptosporidium* infection, and the findings provide a novel view for implementing control strategies against *Cryptosporidium* infection.

**Graphic Abstract:**

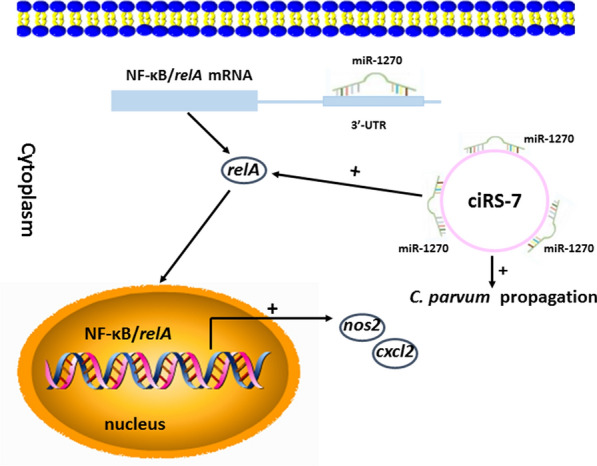

**Supplementary Information:**

The online version contains supplementary material available at 10.1186/s13071-021-04739-w.

## Background

Cryptosporidiosis, caused by the protozoan zoonotic parasite *Cryptosporidium*, is a ubiquitous gastrointestinal illness of humans and many domestic and wild animals. Asymptomatic to severe clinical outcomes can occur because of *Cryptosporidium* infection, and diarrhea is the most common sequela. *Cryptosporidium* has been listed as the fifth leading cause of diarrhea in children < 5 years of age by the Global Burden of Disease Study (GBD) 2016 and has been identified as the second leading cause of diarrhea-associated mortality in children of this age by the Global Burden of Disease Study (GBD) 2015 [1, 2]. Apart from diarrhea, malabsorption and growth impairment are also found in children in less developed countries and impoverished nations due to chronic infection with *Cryptosporidium* [3, 4]. Furthermore, cryptosporidiosis is an important food- and waterborne disease that is responsible for > 8 million cases of foodborne illness annually with > 524 waterborne outbreaks documented from the start of the last century to 2016 [2, 4–6]. The serious consequences of cryptosporidiosis are underestimated and underreported owing to commonly asymptomatic carriage in immunocompetent hosts, insufficient attention, and the unavailability of perfect diagnostic procedures for this important disease worldwide. More significantly, vaccines or effective therapeutics to control cryptosporidiosis are still insurmountable challenges. Thus far, only one drug, nitazoxanide, has been proven to treat cryptosporidiosis [7]. However, this drug is only 56–96% effective in immunocompetent hosts, and no efficacy is found in immune-compromised individuals, especially in patients with advanced AIDS [7]. Considering that the severity of cryptosporidiosis is closely associated with host status, especially immunity [7, 8], thoroughly understanding the interaction between hosts and *Cryptosporidium* is essential to develop effectively well-directed control strategies against cryptosporidiosis.

In recent years, noncoding RNAs (ncRNAs), comprising 95% of the total amount of RNA and transcribed from what were previously considered “junk DNA” sequences, have emerged as novel functional molecules or biomarkers in the progression and processes of a variety of diseases [9, 10]. Among these, circular RNA (circRNA), unlike common linear RNAs, is a covalently closed-ring molecule without the 5′-3′ polar covalently closed loop structure or the polycyclic adenylate tail [11, 12]. This unique RNA transcript is resistant to RNase R, and ongoing studies have shown that it can act as a novel key regulator of gene expression [11]. Next-generation RNA sequencing (RNA-seq) of nonpolyadenylated transcriptomes has uncovered > 10,000 different circRNAs in metazoans [11], and aberrant expression of host circRNAs was induced by infections of various pathogens, including bacteria (e.g. *Mycobacterium tuberculosis*, *Chlamydia trachomatis*), viruses (e.g. porcine endemic diarrhea virus, transmissible gastroenteritis virus), and parasites (e.g. *Nosema ceranae*, *Toxoplasma gondii*, *Eimeria necatrix*) [13–18]. Using the RNA-seq technique without RNase R treatment, our group found 104 significantly differentially expressed circRNAs in tracheal tissues of chickens experimentally infected with *C. baileyi*. Pathway enrichment of these differential circRNAs identified several pathways (e.g. amino sugar and nucleotide sugar metabolism, tight junction, and glycerolipid metabolism) associated with cryptosporidiosis [19], suggesting the potentially important roles of these circRNAs during *Cryptosporidium* infection. However, a small number of circRNAs were identified by the RNA-seq technique used in this study, and fake circRNAs were produced by using this technique. Furthermore, the functional mechanisms of circRNAs during *Cryptosporidium* infection were not revealed in this work. To date, > 40 valid *Cryptosporidium* species have been recognized [4]. Among them, *C. parvum* is the most common zoonotic species infecting humans and many animals [20, 21] and is responsible for the majority of foodborne outbreaks [4]. In the present study, the expression profiles of circRNAs were investigated in HCT-8 cells infected with *C. parvum* using a microarray assay with RNA samples after treatment with RNase R, and the role and mechanism of a significantly upregulated circRNA, ciRS-7, were preliminarily studied.

## Methods

### Cells, parasites, and *in vitro* infection model

The human ileocecal adenocarcinoma (HCT-8) cell line was obtained from JENNIO Biological Technology (Guangzhou, China) and cultured in RPMI 1640 medium supplemented with 10% fetal bovine serum at 37 °C with 5% CO_2_. *C. parvum* oocysts were initially collected from neonatal dairy calves with clinical symptoms of diarrhea in Yangling of Shaanxi Province, northwestern China, identified as the *C. parvum* IIdA19G1 subtype using molecular tools targeting the 18S rRNA gene and 60 kDa glycoprotein (*gp60*) gene (data not shown) and propagated in neonatal dairy calves under conditions with no specific pathogens. The *C. parvum* oocysts were purified by Sheather’s sugar flotation and cesium chloride density gradient centrifugation, resuspended in phosphate-buffered saline solution (PBS) supplemented with 100 U/ml penicillin, 100 μg/ml streptomycin, and 0.25 μg/ml amphotericin B solutions, and stored at 4 °C. An *in vitro* infection model of *C. parvum* was established according to our previous study [21], using an infection ratio of 2–10:1 between oocysts and HCT-8 cells.

### Total RNA extraction and circRNA microarray analysis

Total RNA was isolated using the TRIzol reagent (Invitrogen, Gaithersburg, MD, USA) and purified by using a NucleoSpin® RNA clean-up kit (MACHEREY-NAGEL, Dueren, Germany) according to the manufacturer’s instructions. The purity and integrity of the total RNA were determined using a ND-1000 Spectrophotometer (Thermo Fisher Scientific Inc., Wilmington, DE, USA) and 1% agarose gel electrophoresis, respectively. The qualified total RNA was treated with RNase R (Epicentre, Madison, WI, USA) to remove linear RNA and then transcribed into double-strand cDNA using an Ambion® WT Expression Kit (Thermo Fisher Scientific Inc., Wilmington, DE, USA). The second strand of cDNA was used as the template to synthesize the cRNA, which was then purified by an RNA purification column to remove the salt, enzyme, and other reagents in the reaction and transcribed into cDNA utilizing random primers. The transcribed cDNA was labeled with Cy3-dCTP and hybridized with the circRNA Human Gene Expression Microarray Array V2.0 (4 × 180 K) (CapitalBio Technology Corp., Beijing, China). The hybridization arrays were washed and scanned by using an Agilent Scanner (G2565CA), and Agilent Feature Extraction v10.7 software was used to analyze and extract raw data from scanned images. The extracted data were normalized and processed by using Agilent GeneSpring software. The differentially expressed circRNAs during *C. parvum* infection showing statistical significance were filtered by fold change > 2.0 and *P*-value ≤ 0.05.

### Quantitative real-time polymerase chain reaction (qRT-PCR)

Total RNA samples were isolated using TRIzol reagent (Beijing CoWin Biotech Co., Ltd., Beijing, China) and then transcribed into cDNA for RNA and miRNA verification by using the Primescript™ reagent kit with gDNA Eraser (Takara Biomedical Technology, Dalian, China) and the Mir-X miRNA First-Strand Synthesis Kit (Takara Biomedical Technology, Dalian, China) according to the manufacturer's instructions. qRT-PCRs were performed by using SBRY Green PCR Master Mix (Beijing CoWin Biotech Co., Ltd., Beijing, China), with *gapdh* and *u6* used as internal references for RNAs and miRNAs, respectively. Experiments were carried out in triplicate, and the relative expression of each RNA was calculated using the 2^−ΔΔ*Ct*^ method. The primer sequences used in the present study are listed in Additional file 1: Table S1.

### Cell transfection

Small interfering RNA (siRNA) against ciRS-7 (si-ciRS-7) and scramble RNA (si-control) was purchased from RiboBio Co., Ltd. (Guangzhou, China), and the ciRS-7 overexpression vector was constructed by using the pcDNA3.1( +) plasmid (Invitrogen, Gaithersburg, MD, USA). miR-1270 mimics and inhibitors with their negative controls were obtained from GenePharma (Shanghai, China). For transfection, Lipofectamine 2000 reagent (Invitrogen, Gaithersburg, MD, USA) was used following the manufacturer’s protocols. The sequences of siRNA and miRNA mimics and inhibitors are presented in Additional file 2: Table S2.

### Western blotting

The protein samples were extracted by RIPA lysis buffer (Beijing Applygen Technologies Co., Ltd., Beijing, China) supplemented with protease and phosphatase inhibitors (Beijing Solarbio Science & Technology Co., Ltd., Beijing, China) and denatured in boiling water for 10 min. The denatured proteins were separated by sodium dodecyl sulfate polyacrylamide gel electrophoresis (SDS-PAGE) and transferred to polyvinylidene difluoride (PVDF) membranes (Millipore, Billerica, USA). The membranes were blocked in PBST (0.05% Tween-20) solution containing 5% nonfat milk for 1 h to remove nonspecific binding. Following overnight incubation with the primary antibodies anti-RELA (1:1000, ABclonal, Wuhan, China) or anti-GAPDH (1:500,000, ABclonal, Wuhan, China) at 4 °C, the membranes were incubated with a horseradish peroxidase (HRP)-conjugated secondary antibody (1:2000, Shanghai Sangon Biotech, Shanghai, China) at room temperature for 1 h. An enhanced chemiluminescence (ECL) system was applied to visualize protein bands, and ImageJ software was used to quantify protein expression levels.

### Dual luciferase reporter assay

The plasmids of wild-type ciRS-7 (ciRS-7-WT) and *relA* (*relA*-WT) and mutant ciRS-7 (ciRS-7-MUT) and *relA* (*relA*-MUT) were designed and inserted into pmirGLO vectors (Promega, Madison, WI, USA). Each constructed plasmid was cotransfected with miR-1270 mimics or control mimics into HCT-8 cells by using Lipofectamine 2000 reagent (Invitrogen, Gaithersburg, MD, USA). Luciferase activities were measured after 48 h of incubation by using a multimode reader assay (Spark, Tecan, Männedorf, Switzerland).

### Statistical analysis

Results were presented as the mean ± SD. Data from different groups were compared by using one-way ANOVA. All statistical analyses were processed using GraphPad PRISM 6.07 software (San Diego, CA, USA) or IBM SPSS statistics version 22.0 software (Armonk, NY, USA). A *P*-value < 0.05 was considered statistically significant.

## Results

### *C. parvum* infection induced significantly aberrant expression of circRNA profiles in HCT-8 cells

**Fig. 1:**
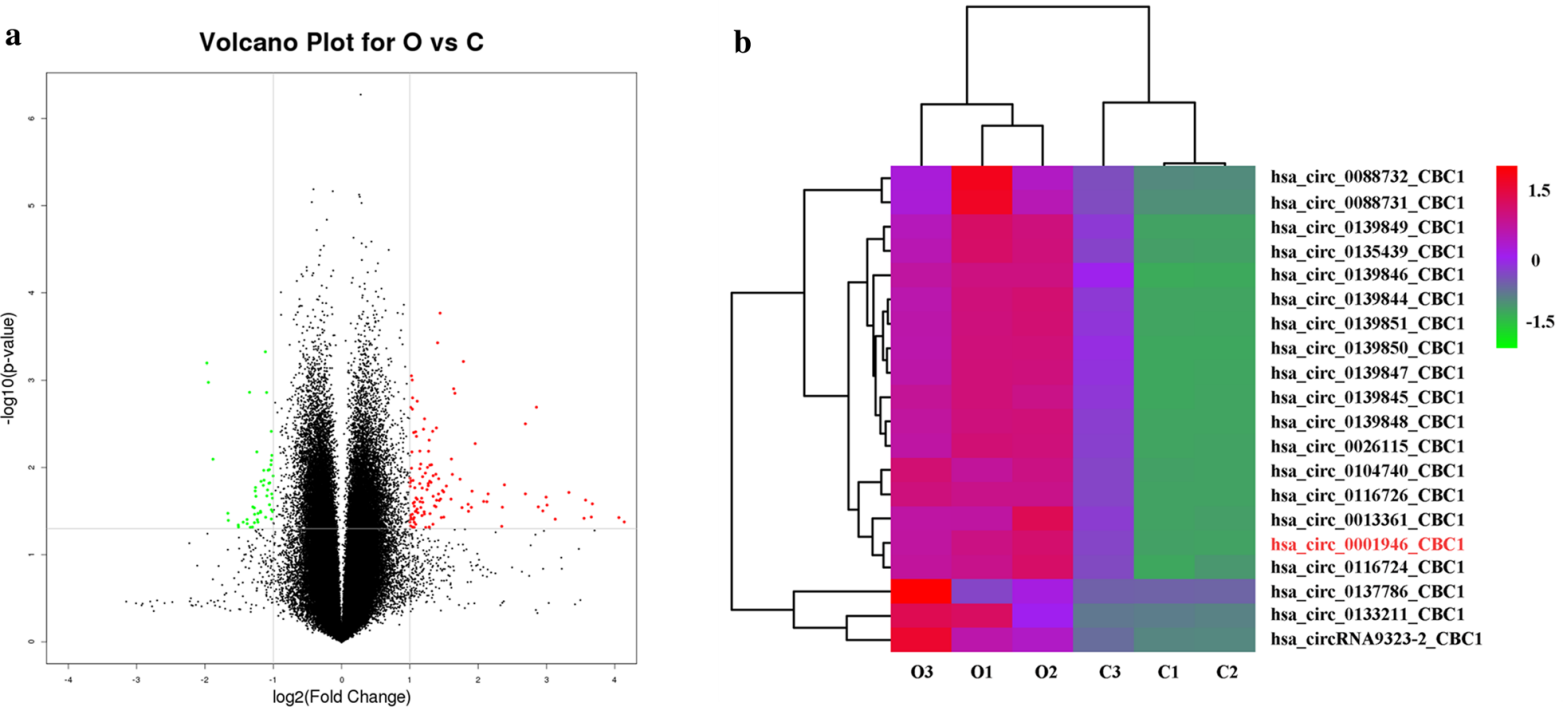
Microarray analysis of differentially expressed circRNAs in HCT-8 cells infected with *Cryptosporidium parvum* IIdA19G1 subtype. **a** Volcano plots showed differently expressed circRNAs in response to *C. parvum* infection. Significantly differentially expressed circRNAs with a fold change > 2.0 and *P* ≤ 0.05 are shown in red (upregulated) or green (downregulated) dots. **b** Heat maps presented the top 20 significantly upregulated circRNAs in HCT-8 cells in response to *C. parvum* infection

Following *Cryptosporidium* infection for 24 h, a total of 178 (including 128 up- and 50 downregulated) circRNAs were found to be significantly differentially expressed (DE) in HCT-8 cells with a fold change > 2.0 and *P* ≤ 0.05 (Fig. 1a, b and Additional file 3: Table S3). GO and KEGG pathway analysis of genes producing DE circRNAs indicated that these DE circRNAs were involved in several important biological processes (e.g. leukocyte chemotaxis, chemotaxis, and cellular response to epinephrine stimulus), molecular functions (e.g. adrenergic receptor binding, adenyl ribonucleotide binding, and adenyl nucleotide binding) (Additional file 4: Table S4 and Additional file 6: Figure S1), and vital signaling pathways (e.g. ECM-receptor interaction, focal adhesion, and purine metabolism) (Additional file 5: Table S5 and Additional file 7: Figure S2) during host-*Cryptosporidium* interaction.

### The expression of ciRS-7 was induced by *C. parvum* infection in HCT-8 cells

**Fig. 2:**
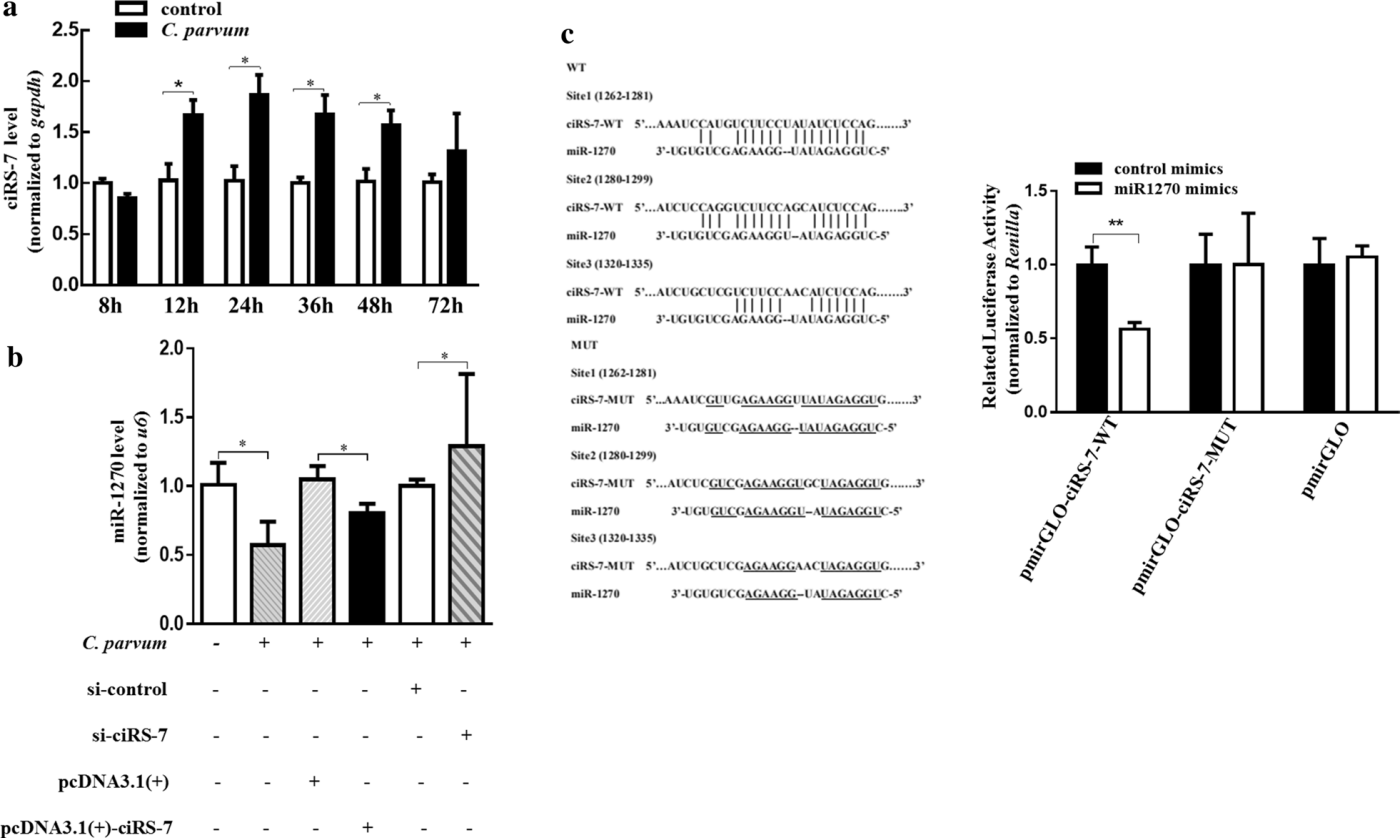
CiRS-7 acted as a sponge for miR-1270 in HCT-8 cells during *C. parvum* infection. **a** Time-dependent expression of ciRS-7 levels in HCT-8 cells during *C. parvum* infection. **b** The miR-1270 levels in HCT-8 cells transfected with pcDNA3.1( +)-ciRS-7 plasmid or si-ciRS-7 and exposed to *C. parvum* infection for 24 h. **c** The luciferase activity in HCT-8 cells co-transfected with miR-1270 mimics and pmirGLO-ciRS-7-WT or pmirGLO-ciRS-7-MUT. Left represents the putative binding site between ciRS-7 and miR-1270 predicted by starBase v2.0, and right represents the luciferase reporter assay for ciRS-7-WT or ciRS-7-MUT in HCT-8 cells co-transfected with miR-1270 mimics. The data represent the mean ± SD for three independent experiments. *P < 0.05, **P < 0.01

Among 128 significantly upregulated circRNAs, a previously well-studied circRNA, hsa_circ_0001946_CBC1 (ciRS-7, also known as CDR1as), was remarkably upregulated by nearly eightfold following *C. parvum* infection for 24 h in microarray analysis (Additional file 3: Table S3). qRT-PCR confirmed the microarray results and found continuously increased expression of ciRS-7 in HCT-8 cells from 12-h post-infection (pi) to 48 hpi (Fig. 2a), suggesting the potential role of this circRNA during *C. parvum* infection.

### CiRS-7 acted as a sponge of miR-1270 in HCT-8 cells following *C. parvum* infection

Considering the involvement of ciRS-7 in many physiological or pathological processes via binding to specific miRNAs [22–24], we predicted 22 potential miRNA targets of ciRS-7 by using starBase v2.0 [25]. By constructing ciRS-7-miRNA networks (Additional file 8: Figure S3) with Cytoscape software [26], we identified a miRNA, miR-1270, which was one of the most highly matched miRNAs of ciRS-7 [27] and acted as a sponge to regulate the progression of ovarian cancer [28], bladder cancer [29], and hepatocellular carcinoma [27]. In the present study, the expression of miR-1270 was significantly decreased in HCT-8 cells infected with *C. parvum* at 24 hpi (Fig. 2b), and the dual-luciferase reporter assay showed that cotransfection of pmirGLO-ciRS-7-WT luciferase reporter vector with miR-1270 mimics, but not pmirGLO-ciRS-7-MUT vector, markedly reduced the ciRS-7-regulated luciferase activity in HCT-8 cells (Fig. 2c). Furthermore, overexpression of ciRS-7 conspicuously decreased miR-1270 expression levels in *C. parvum*-infected HCT-8 cells, while knockdown of ciRS-7 using siRNA targeting ciRS-7 had the reverse effect (Fig. 2b and Additional file 9: Figure S4). These results demonstrated that ciRS-7 might sponge miR-1270 in HCT-8 cells in response to *C. parvum* infection.

### *RelA* was a direct target of miR-1270 in HCT-8 cells following *C. parvum* infection

**Fig. 3:**
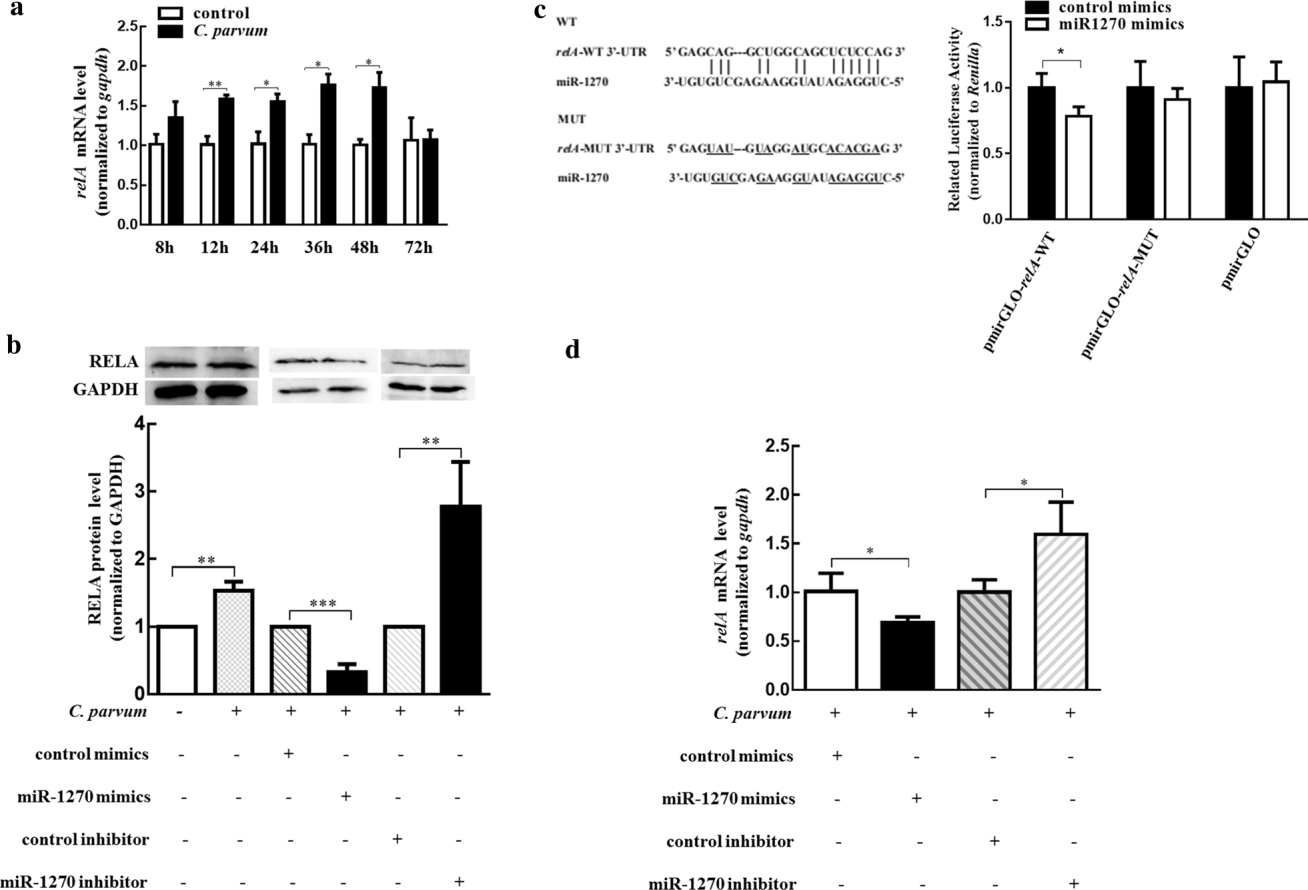
*RelA* was a direct target of miR-1270 in HCT-8 cells following *C. parvum* infection. **a** Time-dependent expression of *relA* mRNA levels in HCT-8 cells during *C. parvum* infection. **b** Protein levels of RELA in HCT-8 cells transfected with miR-1270 mimics or inhibitor and exposed to *C. parvum* infection for 24 h. **c** The luciferase activity in HCT-8 cells co-transfected with miR-1270 mimics and pmirGLO-*relA*-WT or pmirGLO-*relA*-MUT. Left represents the putative binding site between the 3′-UTR of *relA* and miR-1270 predicted by starBase v2.0, and right represents the luciferase reporter assay for *relA*-WT or *relA*-MUT in HCT-8 cells co-transfected with miR-1270 mimics. **d** The mRNA levels of *relA* in HCT-8 cells transfected with miR-1270 mimics or inhibitor and exposed to *C. parvum* infection for 24 h. The data represent the mean ± SD for three independent experiments. *P < 0.05, **P < 0.01, ***P < 0.001

It is well known that miRNAs can regulate various biological processes through perfect or partial base pairing of the 3′-UTR regions of the target mRNAs [30]. To better understand the potential function of miR-1270 in HCT-8 cells following *C. parvum* infection, *relA* (also known as *p65*), a subunit of the NF-κB signaling pathway which was proven to be a key pathway to prevent epithelial cell apoptosis, thus benefiting parasite propagation [31], was predicted to be a potential target of miR-1270 by using the starBase v2.0 database. In our study, the mRNA level of *relA* was significantly upregulated during *C. parvum* infection from 12 to 48 hpi (Fig. 3a), and the protein level was remarkably increased in HCT-8 cells exposed to *C. parvum* for 24 h (Fig. 3b). A dual-luciferase reporter assay found that the luciferase activity of the pmirGLO-*relA*-WT reporter vector was significantly decreased by miR-1270 mimics (Fig. 3c). Moreover, both qRT-PCR (Fig. 3d) and western blot (Fig. 3b) assays showed that *relA* expression was decreased by miR-1270 mimics but increased by miR-1270 inhibitor in HCT-8 cells infected with *C. parvum*. Thus, miR-1270 might directly regulate *relA* mRNA and RELA protein expression during *C. parvum* infection in HCT-8 cells.

### CiRS-7 regulated *relA* expression by sponging miR-1270 in HCT-8 cells following *C. parvum* infection

**Fig. 4:**
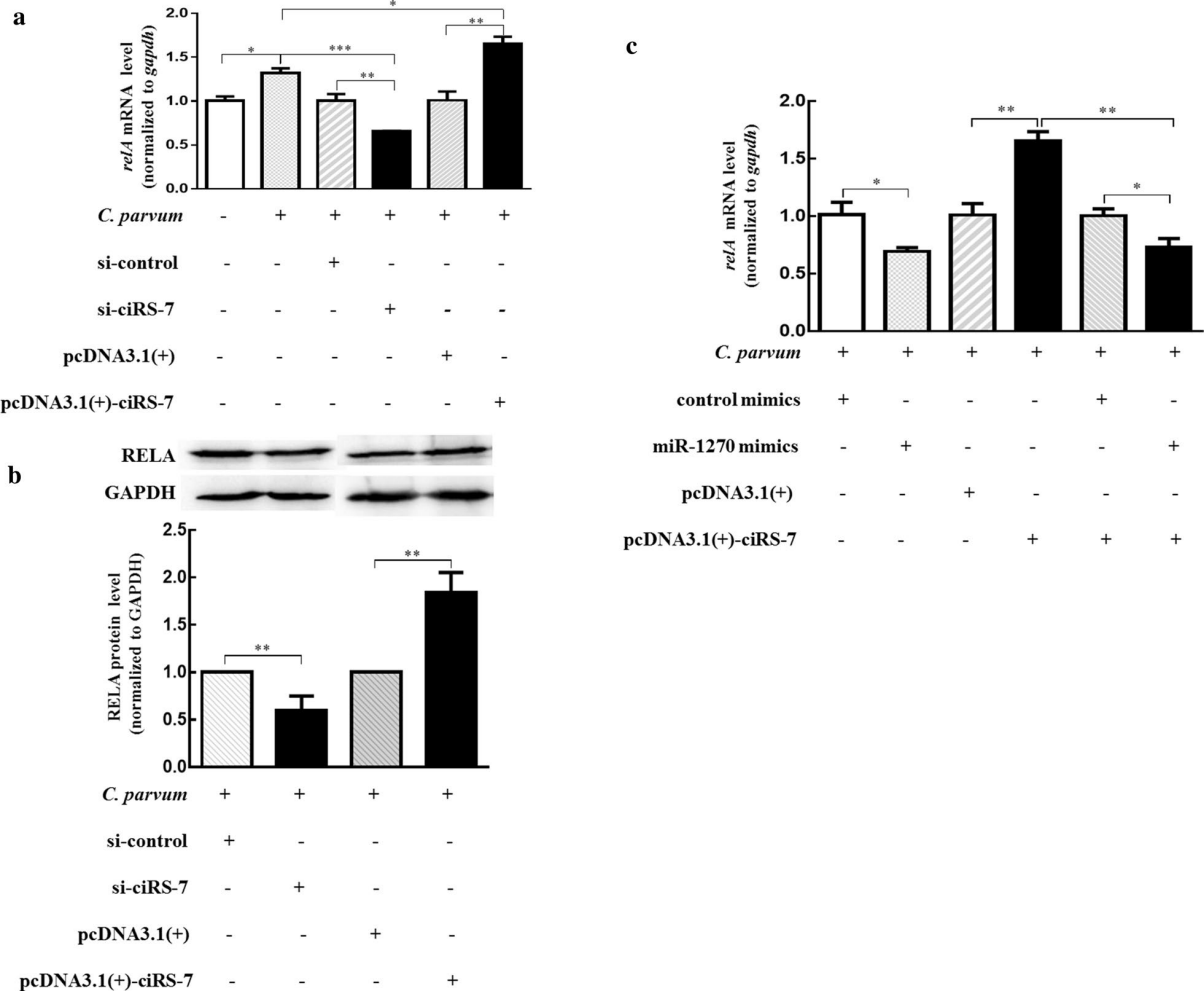
CiRS-7 promoting *relA* expression in *C. parvum*-infecting HCT-8 cells via sponging miR-1270. **a**,** b** mRNA (**a**) and protein (**b**) levels of *relA* in HCT-8 cells transfected with pcDNA3.1( +)-ciRS-7 plasmid or si-ciRS-7 and exposed to *C. parvum* infection for 24 h. **c** The mRNA levels of *relA* in HCT-8 cells co-transfected with pcDNA3.1( +)-ciRS-7 plasmid and miR-1270 mimics and exposed to *C. parvum* infection for 24 h. The data represent the mean ± SD for three independent experiments. *P < 0.05, **P < 0.01, ***P < 0.001

CircRNAs act as competing endogenous RNAs (ceRNAs) to sponge miRNAs and consequently regulate the expression of the targeted mRNAs [32]. In the present study, we tested whether ciRS-7 modulated *relA* expression by adsorbing miR-1270 in HCT-8 cells following *C. parvum* infection. We found that the expression pattern of *relA* was similar to that of ciRS-7 and was also significantly increased from 12 to 48 hpi (Fig. 3a). The upregulation of *relA* induced by *C. parvum* was attenuated by silencing of ciRS-7 using siRNA (si-ciRS-7), while overexpression of ciRS-7 enhanced the effect of *C. parvum* on the *relA* gene (Fig. 4a, b). Cotransfection of pcDNA3.1( +)-ciRS-7 vector and miR-1270 mimics into HCT-8 cells showed that the upregulated effect of ciRS-7 on *relA* expression during *C. parvum* infection was reversed by miR-1270 mimics (Fig. 4c). These results indicated that ciRS-7 regulated *relA* by targeting miR-1270 during *C. parvum* infection.

### CiRS-7 stimulated the NF-κB pathway by sponging miR-1270 in HCT-8 cells following *C. parvum* infection

**Fig. 5:**
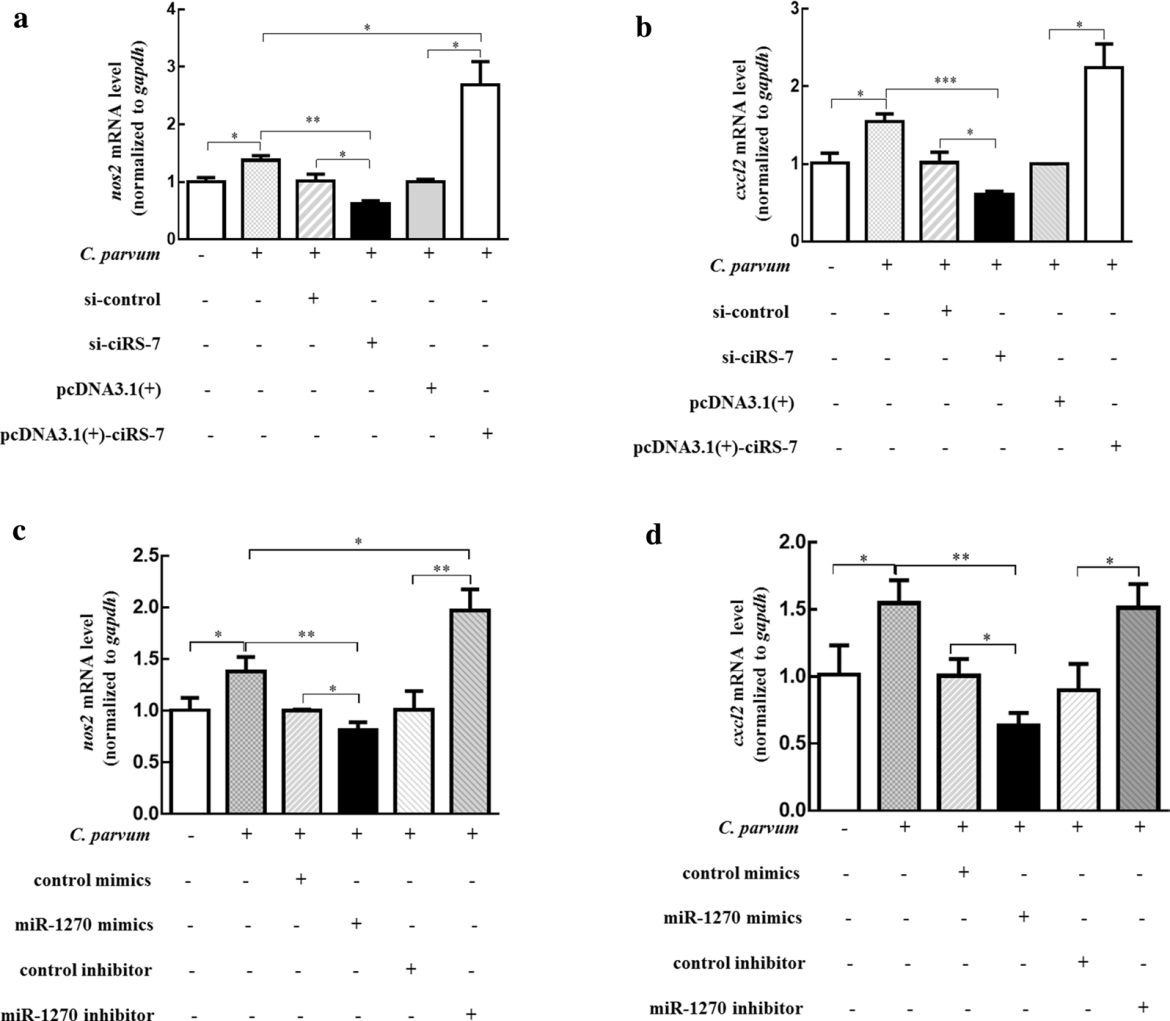
The impact of ciRS-7/miR-1270 axis on the expression of NF-κB signal downstream molecules of *nos2* (**a**, **c**) and *cxcl2* (**b**, **d**) in HCT-8 cells following *C. parvum* infection. HCT-8 cells were transfected with pcDNA3.1( +)-ciRS-7 plasmid or si-ciRS-7 (**a**, **b**) and transfected with miR-1270 mimics or inhibitor (**c**, **d)**, followed by exposing to *C. parvum* infection for 24 h. The mRNA levels of *nos2* (**a**, **c**) and *cxcl2* (**b**, **d**) were detected by qRT-PCR. The data represent the mean ± SD for three independent experiments. *P < 0.05, **P < 0.01, ***P < 0.001

**Fig. 6:**
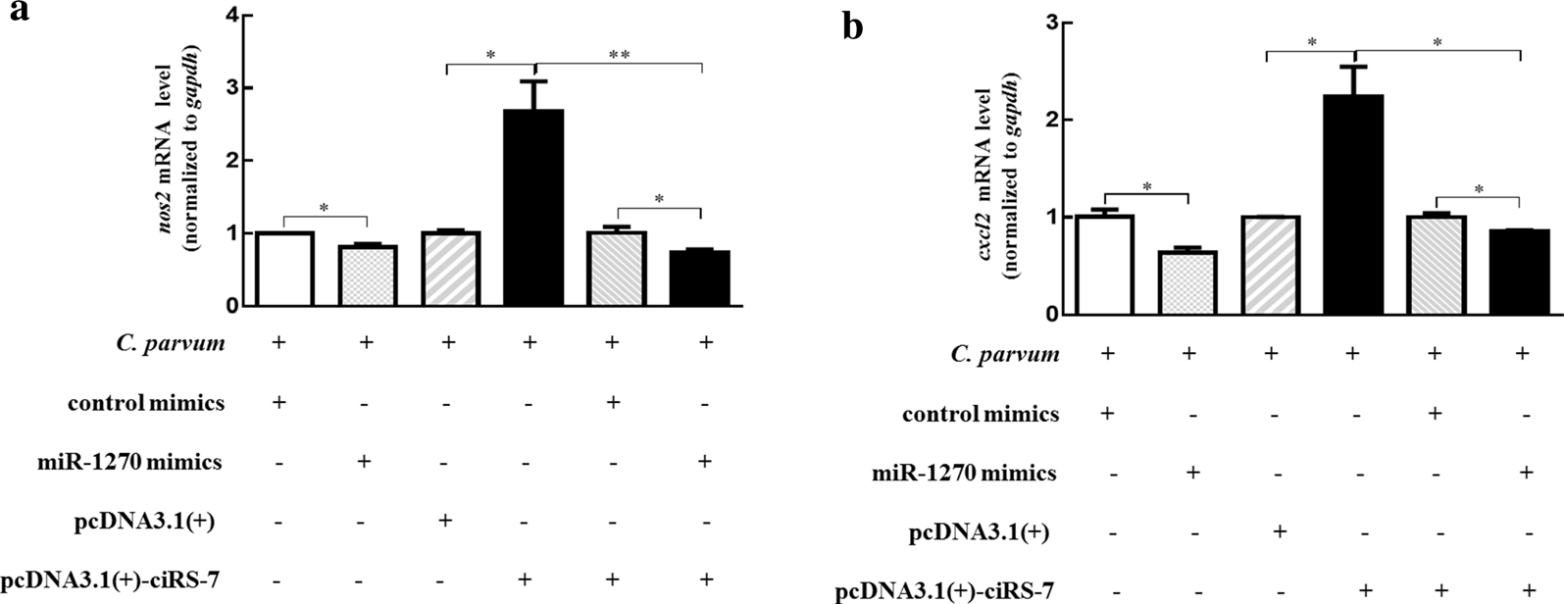
The mRNA levels of *nos2* (**a**) and *cxcl2* (**b**) in HCT-8 cells co-transfected with pcDNA3.1( +)-ciRS-7 plasmid and miR-1270 mimics, followed by exposing to *C. parvum* infection for 24 h. The data represent the mean ± SD for three independent experiments. *P < 0.05, **P < 0.01

Given that ciRS-7 affected the expression of the subunit *relA* within the NF-κB signaling pathway by sponging miR-1270 during *C. parvum* infection, we asked whether the ciRS-7/miR-1270 axis regulated the NF-κB signaling pathway. To address this question, we investigated the expression of two important downstream molecules of the NF-κB signaling pathway, *nos2* and *cxcl2*, which were both upregulated during *C. parvum* infection [33]. In our study, the expression of *nos2* and *cxcl2* was remarkably increased in HCT-8 cells infected with *C. parvum* at 24 hpi (Fig. 5). Overexpression of ciRS-7 markedly increased the expression of two genes in HCT-8 cells infected with *C. parvum*, while knockdown of ciRS-7 significantly decreased their expression (Fig. 5a, b). Notably, the effect of miR-1270 on the expression of *nos2* and *cxcl2* was exactly the opposite of that of ciRS-7 (Fig. 5c, d), and miR-1270 could attenuate the induction effect of ciRS-7 on *nos2* and *cxcl2* (Fig. 6a, b), suggesting that ciRS-7 was implicated in activating the NF-κB pathway by interacting with miR-1270 during *C. parvum* infection.

### Upregulation of ciRS-7 promoted the propagation of *C. parvum* by regulating miR-1270 in HCT-8 cells

**Fig. 7:**
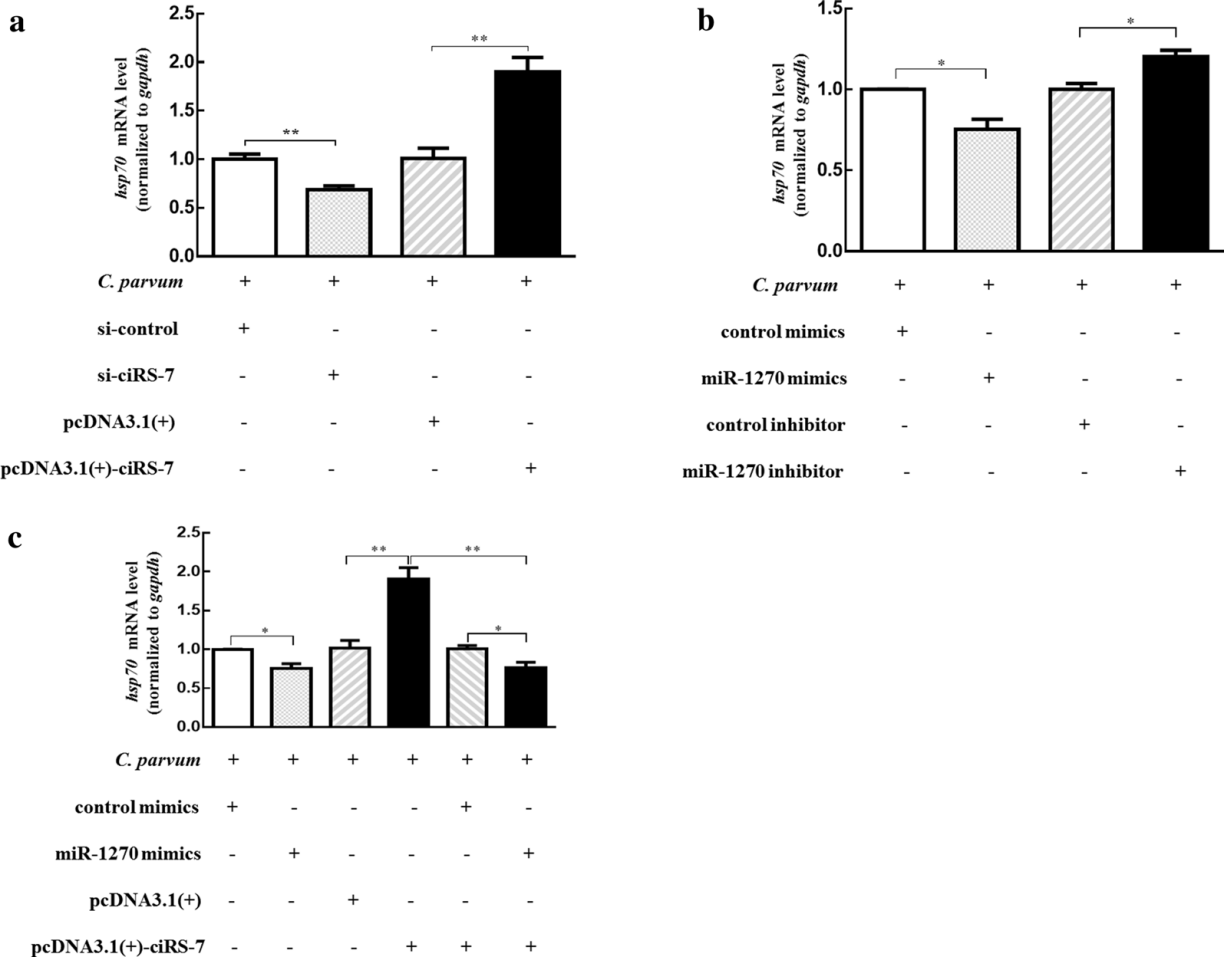
The mRNA levels of *C. parvum hsp70* gene in HCT-8 cells. HCT-8 cells were transfected with pcDNA3.1( +)-ciRS-7 plasmid or si-ciRS-7 (**a**), transfected with miR-1270 mimics or inhibitor (**b**), and co-transfected with pcDNA3.1( +)-ciRS-7 plasmid and miR-1270 mimics (**c**), followed by exposure to *C. parvum* infection for 24 h. The data represent the mean ± SD for three independent experiments. *P < 0.05, **P < 0.01

Activation of the NF-κB signaling pathway significantly affects the propagation of *C. parvum* [31, 33]. Therefore, we studied the role of the ciRS-7/miR-1270 axis in determining the infection burden of *C. parvum* in HCT-8 cells. The mRNA level of the *hsp70* gene of *C. parvum* was enhanced in HCT-8 cells transfected with pcDNA3.1( +)-ciRS-7 compared with control groups with pcDNA3.1( +) (Fig. 7a and Additional file 10: Figure S5), and significantly decreased expression of the *hsp70* gene of *C. parvum* was detected in HCT-8 cells transfected with si-ciRS-7 (Fig. 7a). On the other hand, miR-1270 mimics markedly decreased the expression level of the *C. parvum hsp70* gene (Fig. 7b), but the opposite effect on the infection burden was detected in HCT-8 cells by knockdown of miR-1270 with an inhibitor (Fig. 7b). Furthermore, cotransfection assay showed that the upregulated effect of ciRS-7 on parasite burden was markedly suppressed by miR-1270 mimics (Fig. 7c), indicating that ciRS-7 promoted *C. parvum* propagation by regulating miR-1270 expression.

## Discussion

In recent years, circRNAs, a novel RNA class, are being recognized to function in many kinds of physiological and pathological processes by regulating gene expression at the transcriptional and posttranscriptional levels [34–36]. Significantly aberrant expression of host circRNAs at the genome-wide level was also found during infections with a limited number of parasites [13, 18, 37], including *Cryptosporidium* [19]; however, the functions of these DE circRNAs are still unknown. In the present study, we performed microarray analysis of DE circRNAs in HCT-8 cells infected with the important zoonotic *C. parvum* and identified a total of 178 circRNAs (including 128 upregulated and 50 downregulated) that were abnormally expressed. Furthermore, we found that upregulated ciRS-7 promoted the *in vitro* propagation of *Cryptosporidium* by acting as a miRNA sponge.

ciRS-7, originating from cerebellar degeneration-related protein 1 antisense transcript (CDR1AS), is the most well-known and widely studied circRNA [38, 39]. This circular ncRNA molecule serves as a regulator in many diseases, including many cancers (e.g. breast cancer, colorectal cancer, bladder cancer, esophageal cancer, gastric cancer, hepatocellular cancer, lung cancer, pancreatic cancer, ovarian cancer), gliomas, diabetes, myocardial infarction, atherosclerosis, osteoarthritis, pulmonary fibrosis, osteonecrosis of the femoral head, myocardial infarction, and neurodegenerative diseases (e.g. Alzheimer's disease) [38–41]. In our study, using a *C. parvum*-infected HCT-8 cell model, ciRS-7 was found to be upregulated at 24 hpi by microarray analysis and at 12–48 hpi by qRT-PCR tests. Interference of ciRS-7 expression in HCT-8 cells by using siRNA reduced the parasite burden of *C. parvum*, and transfection of an overexpression (pcDNA3.1( +)-ciRS-7) plasmid into HCT-8 cells elevated the parasite burden. These results indicate that ciRS-7 could promote the propagation of *C. parvum* in HCT-8 cells through certain functional mechanisms.

To date, several functions of circRNAs have been identified, including acting as miRNA sponges that competitively bind to miRNAs to block miRNA-mRNA interactions, docking in the active sites of RNA-binding proteins (RBPs) to activate these RBPs, modulating mRNA stability, and serving as translation templates [35, 36]. Among these, sponging miRNA is a well-known regulatory mechanism for circRNAs, although the general feature of circRNAs blocking the miRNA silencing effect remains controversial [18, 42]. In the present study, using the online tool starBase, miR-1270 was predicted to be a potential miRNA target of ciRS-7. Previous studies reported that miR-1270 served as a sponge of ciRS-7 involved in the progression of hepatocellular carcinoma [27] and cisplatin resistance in ovarian cancer and bladder cancer [28, 29]. The luciferase reporter assay validated the interaction between ciRS-7 and miR-1270 in HCT-8 cells in our study. *C. parvum* infection significantly reduced the expression level of miR-1270 in HCT-8 cells. Overexpression of ciRS-7 also decreased miR-1270 in HCT-8 cells infected with *C. parvum*, while knockdown of ciRS-7 rescued miR-1270 expression in this *in vitro* infection model, further confirming the sponging effect of ciRS-7 on miR-1270 during *C. parvum* infection.

Advances in the knowledge of cryptosporidiosis showed that both innate and adaptive immune responses contributed to clear infection and to prevent reinfection of *Cryptosporidium*, and innate immunity provided a crucially early defense and a key element to activate the adaptive immune system [7, 43]. Although the initiating mechanisms for cellular innate inflammatory responses during *Cryptosporidium* infection are still poorly understood, the Toll-like receptors (TLRs) (especially TLR2 and TLR4) and the propagation of signaling cascades induced by these TLRs have been reported as important innate elements against *Cryptosporidium* infection [43, 44]. *C. parvum* infection triggered activation of the host NF-κB signaling pathway, a key factor in eliciting inflammatory responses and intracellular survival signals in host epithelial cells during exogenous pathogen infection, but this intrinsic defensive signal was found to be monitored by *C. parvum* to successfully propagate and survive [31, 43]. In our study, we detected continuously high levels of *relA* mRNA from 12 to 48 hpi in HCT-8 cells infected with *C. parvum*, and a significantly high protein level was also found at 24 hpi. Furthermore, *nos2* and *cxcl2*, two important downstream signaling molecules of the NF-κB signaling pathway, were highly expressed, indicating activation of the NF-κB signaling pathway in HCT-8 cells infected with *C. parvum*. Emerging evidence has revealed the significant role of ncRNAs responsible for intricately controlling the innate immunity against *Cryptosporidium* infection, including miRNAs and the long noncoding RNAs (lncRNAs) [33, 45]. Recently, circRNAs have been identified as key actors in regulating the innate immune responses against viral and bacterial infection [34–36]. We therefore tested the regulatory effect of ciRS-7 on the NF-κB signaling during infection with *C. parvum*. Overexpression of ciRS-7 increased the expression of RELA (mRNA and protein) and downstream *nos2* and *cxcl2* mRNA levels in infected HCT-8 cells, and vice versa for knockdown of ciRS-7. Considering our finding that ciRS-7 acted as a miRNA sponge for miR-1270 during infection of *C. parvum*, we asked whether ciRS-7 regulated the NF-κB signaling by sponging miR-1270. We confirmed the interaction between miR-1270 and *relA* by using a luciferase reporter assay, and in a *C. parvum*-infected HCT-8 cell model, the inhibitory effect of miR-1270 on the expression of *relA* and downstream *nos2* and *cxcl2* was demonstrated. Notably, cotransfection of miR-1270 mimics and ciRS-7 overexpression vector inhibited the expression of these genes and the burden of *C. parvum* in HCT-8 cells. These results suggested that ciRS-7 sponging miR-1270 to regulate the NF-κB signaling pathway would promote *Cryptosporidium* infection. However, activation of the NF-κB signaling pathway would lead to the induction of essential proinflammatory molecules for the host antipathogen response [46, 47], although previous studies have shown that the NF-κB signaling limited the apoptosis of infected cells to facilitate the growth and maturation of *C. parvum* at the early infection stage [31, 48]. To address the potential effect of ciRS-7 on host intrinsic immune defense apoptosis during *C. parvum* infection, we investigated the expression of *Bcl-2* (Additional file 11: Figure S6), the central regulator of apoptosis. Upregulation of *Bcl-2* was detected from 12 to 48 hpi during *C. parvum* infection (Additional file 11: Figure S6a). Significantly, overexpression of ciRS-7 further increased the mRNA level of *Bcl-2* at 24 hpi (*P* < 0.05), and silencing of ciRS-7 dramatically decreased the expression of *Bcl-2* at 24 hpi (*P* < 0.05) (Additional file 11: Figure S6b). Upregulation of ciRS-7 promoted *Bcl-2* expression to inhibit apoptosis, which was also reported in osteoarthritis (OA) model cells [49]. During *Crptosporidium* infection, apoptosis was increased in HCT-8 cells with siRNA treatment of the *Bcl-2* gene after *C. parvum* infection, and the percentage of infected cells decreased by 1.4-fold at 24 hpi [50]. These results together with our findings suggested that ciRS-7 may promote the propagation of *C. parvum* in HCT-8 cells by affecting apoptosis. To further test this hypothesis, infected and neighboring cells in the same cultures should be sorted and evaluated for apoptosis in future studies. Moreover, future studies are needed to explore other functions of the ciRS-7/miR-1270 axis to modulate epithelial cell antiparasite defense during *Cryptosporidium* infection.

## Conclusions

*C. parvum* infection altered the expression profiles of circRNAs in HCT-8 cells. Our findings suggested that the ciRS-7/miR-1270 axis may promote the propagation of *C. parvum in vitro* by activating the host cell NF-κB signaling pathway and providing a fundamental basis to develop effective strategies against cryptosporidiosis. Certainly, several limitations exist in our work. For example, previous studies showed that *C. parvum* suppressed apoptosis of directly infected cells by activating the NF-κB signaling in these cells, and it is unclear whether there is an association between the ciRS-7/miR-1270 axis and directly infected cell apoptosis. Do other sponging miRNAs of ciRS-7 exist? Is the regulatory effect of the ciRS-7/miR-1270 axis *in vivo* the same? These scientific questions should be investigated in future studies to comprehensively explain the role of circRNAs during *Cryptosporidium* infection.

## Supplementary Information

**Additional file 1**: **Table S1**. Primers for qRT-PCR used in this study (DOCX 16 KB)

**Additional file 2**: **Table S2**. Sequences of siRNA and miRNA mimics and inhibitors used in this study (DOCX 15 KB)

**Additional file 3**: **Table S3**. Significantly differentially expressed circRNAs between *C. parvum*-infected and noninfected HCT-8 cells (XLSX 95 KB)

**Additional file 4**: **Table S4**. GO analysis of genes producing DE circRNAs (XLSX 386 KB)

**Additional file 5**: **Table S5**. KEGG pathway analysis of genes producing DE circRNAs (XLSX 75 KB)

**Additional file 6**: **Figure S1**. The top 30 significantly enriched terms in GO analysis of genes producing DE circRNAs. Blue bars represent biological process terms. Red bars represent molecular function terms (TIF 2564 KB)

**Additional file 7**: **Figure S2**. The top ten significantly enriched terms in KEGG pathway analysis of genes producing DE circRNAs (TIF 1351 KB)

**Additional file 8**: **Figure S3**. The regulatory network of ciRS-7 and its potential sponging miRNAs. The circular red nodes represent circRNAs, and the circular green nodes represent miRNAs (TIF 602 KB)

**Additional file 9**: **Figure S4**. ciRS-7 and miR-1270 were successfully inhibited or overexpressed in HCT-8 cells. HCT-8 cells were transfected with four siRNAs targeting ciRS-7 (a), pcDNA3.1(+)-ciRS-7 plasmid (b), miR-1270 inhibitor (c), and miR-1270 mimics (d) for 24 h, and the expression levels of ciRS-7 or miR-1270 were analyzed by qRT-PCR. The data represent the mean ± SD of three independent experiments. *P < 0.05, **P < 0.01 (TIF 332 KB)

**Additional file 10**: **Figure S5**. The expression of *Crytposporidium *hsp70 in HCT-8 cells infected with *C. parvum*. a. The Ct values of *Crytposporidium* hsp70 for each HCT-8 cell sample with (O1-O3) or without (C1-C3) *C. parvum* infections obtained from qRT-PCR assays. b. Agarose gels of qRT-PCR assays. Lane M represents DL500 DNA Marker (TIF 379 KB)

**Additional file 11**: **Figure S6**. *Bcl-2* expression in HCT-8 cells following *C. parvum* infection. a Time-dependent expression of *Bcl-2* mRNA levels in HCT-8 cells during *C. parvum* infection. b *Bcl-2 *mRNA levels in HCT-8 cells transfected with pcDNA3.1(+)-ciRS-7 plasmid or si-ciRS-7 and exposed to *C. parvum* infection for 24 h. The data represent the mean ± SD for three independent experiments. *P < 0.05, **P < 0.01 (TIF 299 KB)

## Data Availability

All datasets generated for this study are included in the article/Supplementary Information.

## Abbreviations

HCT-8 cells: HCT-8 human ileocecal adenocarcinoma cells
qRT-PCR: Quantitative real-time polymerase chain reaction
siRNA: Small interfering RNA
NF-κB: Nuclear factor kappa-light-chain-enhancer of activated B cells
PBS: Phosphate buffered saline
TLRs: Toll like receptors
SDS-PAGE: Sodium dodecyl sulfate polyacrylamide gel electrophoresis
PVDF: Polyvinylidene difluoride
ECL: Enhanced chemiluminescence
WT: Wild-type
MUT: Mutant
DE: Differentially expressed
