# Supplementary material for: Circular RNA ciRS-7 affects the propagation of Cryptosporidium parvum in HCT-8 cells by sponging miR-1270 to activate the NF-κB signaling pathway
Source: Parasit Vectors. 2021 May 6;14:238. doi: 10.1186/s13071-021-04739-w (PMC8101149; doi:10.1186/s13071-021-04739-w)
Supplement: Supplementary file 1 — Additional file 1: Table S1. Primers for qRT-PCR used in this study (DOCX 16 KB) [file 13071_2021_4739_MOESM1_ESM.docx]

**Table S1** Primers for qRT-PCR used in this study.

| **Name** |  | **Sequences (5′-3′)** |  |
| --- | --- | --- | --- |
| ciRS-7 | Forward | TCAACTGGCTCAATATCCATGTC |  |
|  | Reverse | ACCTTGACACAGGTGCCAT |  |
| miR-1270  RelA | Forward | CGCTGGAGATATGGAAGAGCTGTGT |  |
|  | Forward | CCCCACGAGCTTGTAGGAAAG |  |
|  | Reverse | CTCATAAGGTGCTGAAGGAGTA |  |
| HSP70 | Forward | AACTTTAGCTCCAGTTGAGAAAGTACTC |  |
|  | Reverse | CATGGCTCTTTACCGTTAAAGAATTCC |  |
| NOS2 | Forward | TGCAGACACGTGCGTTACTCC |  |
|  | Reverse | GGTAGCCAGCATAGCGGATG |  |
| CXCL2 | Forward | AACCGAAGTCATAGCCACAC |  |
|  | Reverse | TTCTGGTCAGTTGGATTTGC |  |
| GAPDH | Forward | TGCACCACCAACTGCTTAGC |  |
|  | Reverse | GGCATGGACTGTGGTCATGAG |  |
| U6 | Forward | GGAACGATACAGAGAAGATTAGC |  |
|  | Reverse | TGGAACGCTTCACGAATTTGCG |  |
