# Supplementary material for: Circular RNA ciRS-7 affects the propagation of Cryptosporidium parvum in HCT-8 cells by sponging miR-1270 to activate the NF-κB signaling pathway
Source: Parasit Vectors. 2021 May 6;14:238. doi: 10.1186/s13071-021-04739-w (PMC8101149; doi:10.1186/s13071-021-04739-w)
Supplement: Supplementary file 2 — Additional file 2: Table S2. Sequences of siRNA and miRNA mimics and inhibitors used in this study (DOCX 15 KB) [file 13071_2021_4739_MOESM2_ESM.docx]

**Table S2** Sequences of siRNA, and miRNA mimics and inhibitor used in this study.

| **Name** |  | **Sequences (5′-3′)** |  |
| --- | --- | --- | --- |
| si-ciRS-7_001  si-ciRS-7_002 |  | TCCAGGGTTTCCGATGGCA |  |
|  |  | ATATCCAGGGTTTCCGATG |  |
| si-ciRS-7_003  si-ciRS-7_004  miR-1270 mimics |  | GCACCTGTGTCAAGGTCTTTT |  |
|  |  | TCTGCAATATCCAGGGTTT |  |
|  | Sense | CUGGAGAUAUGGAAGAGCUGUGU |  |
|  | Antisense | ACAGCUCUUCCAUAUCUCCAGUU |  |
| miR-1270 inhibitor | Sense | ACACAGCUCUUCCAUAUCUCCAG |  |
