# Supplementary figures and images for: Circular RNA ciRS-7 affects the propagation of Cryptosporidium parvum in HCT-8 cells by sponging miR-1270 to activate the NF-κB signaling pathway
Source: Parasit Vectors. 2021 May 6;14:238. doi: 10.1186/s13071-021-04739-w (PMC8101149; doi:10.1186/s13071-021-04739-w)

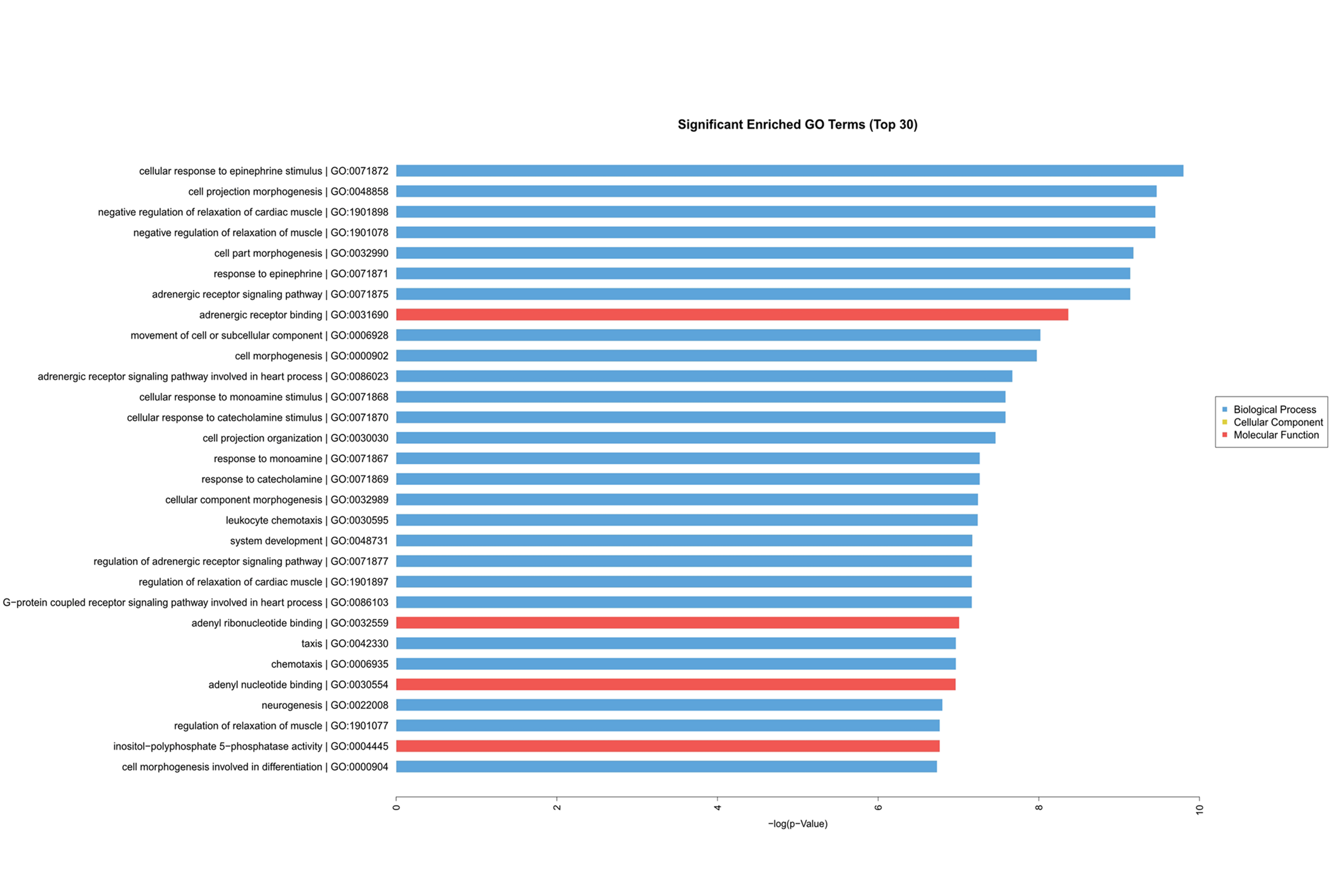

Supplement: Supplementary file 6 — Additional file 6: Figure S1. The top 30 significantly enriched terms in GO analysis of genes producing DE circRNAs. Blue bars represent biological process terms. Red bars represent molecular function terms (TIF 2564 KB) [file 13071_2021_4739_MOESM6_ESM.tif]

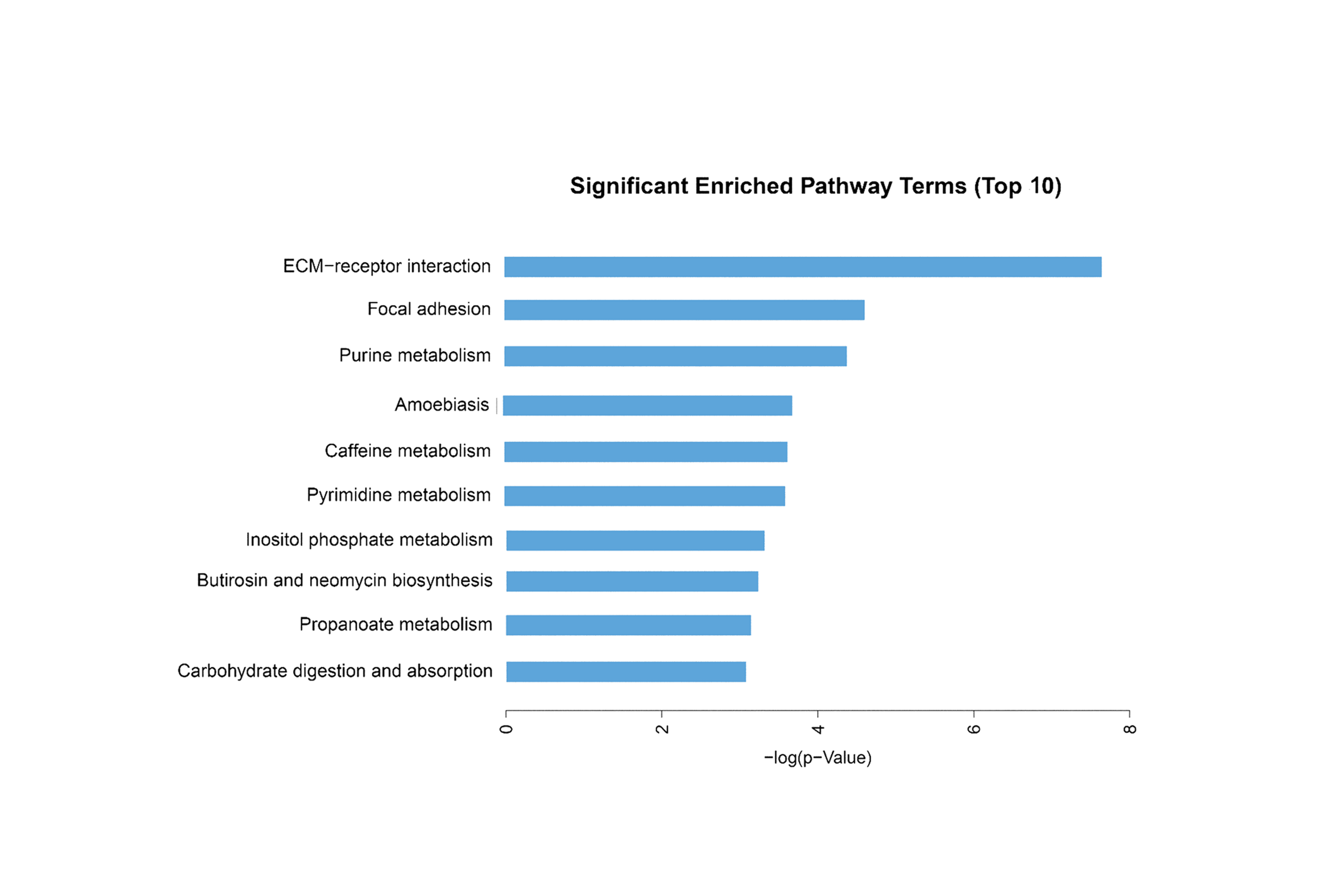

Supplement: Supplementary file 7 — Additional file 7: Figure S2. The top ten significantly enriched terms in KEGG pathway analysis of genes producing DE circRNAs (TIF 1351 KB) [file 13071_2021_4739_MOESM7_ESM.tif]

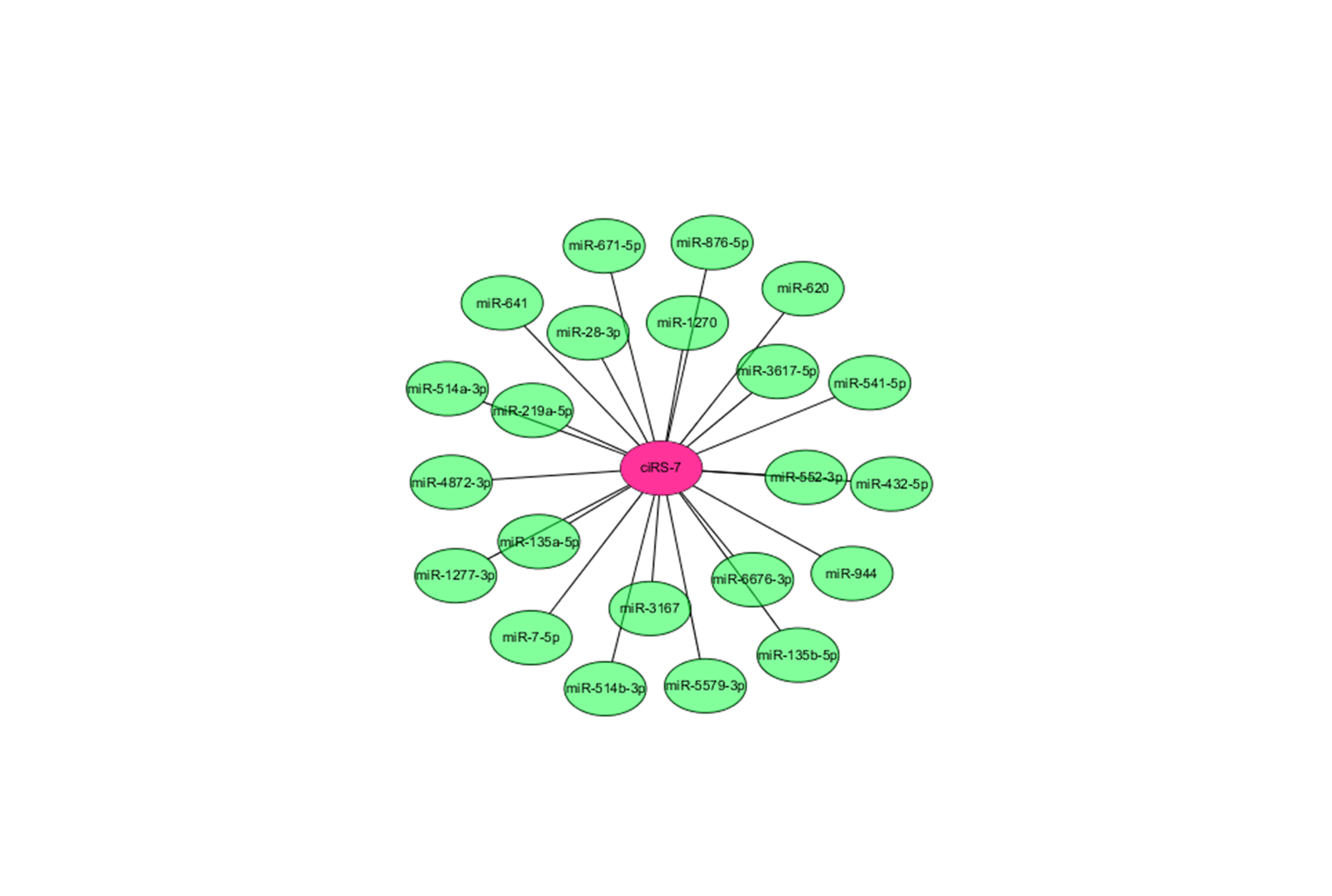

Supplement: Supplementary file 8 — Additional file 8: Figure S3. The regulatory network of ciRS-7 and its potential sponging miRNAs. The circular red nodes represent circRNAs, and the circular green nodes represent miRNAs (TIF 602 KB) [file 13071_2021_4739_MOESM8_ESM.tif]

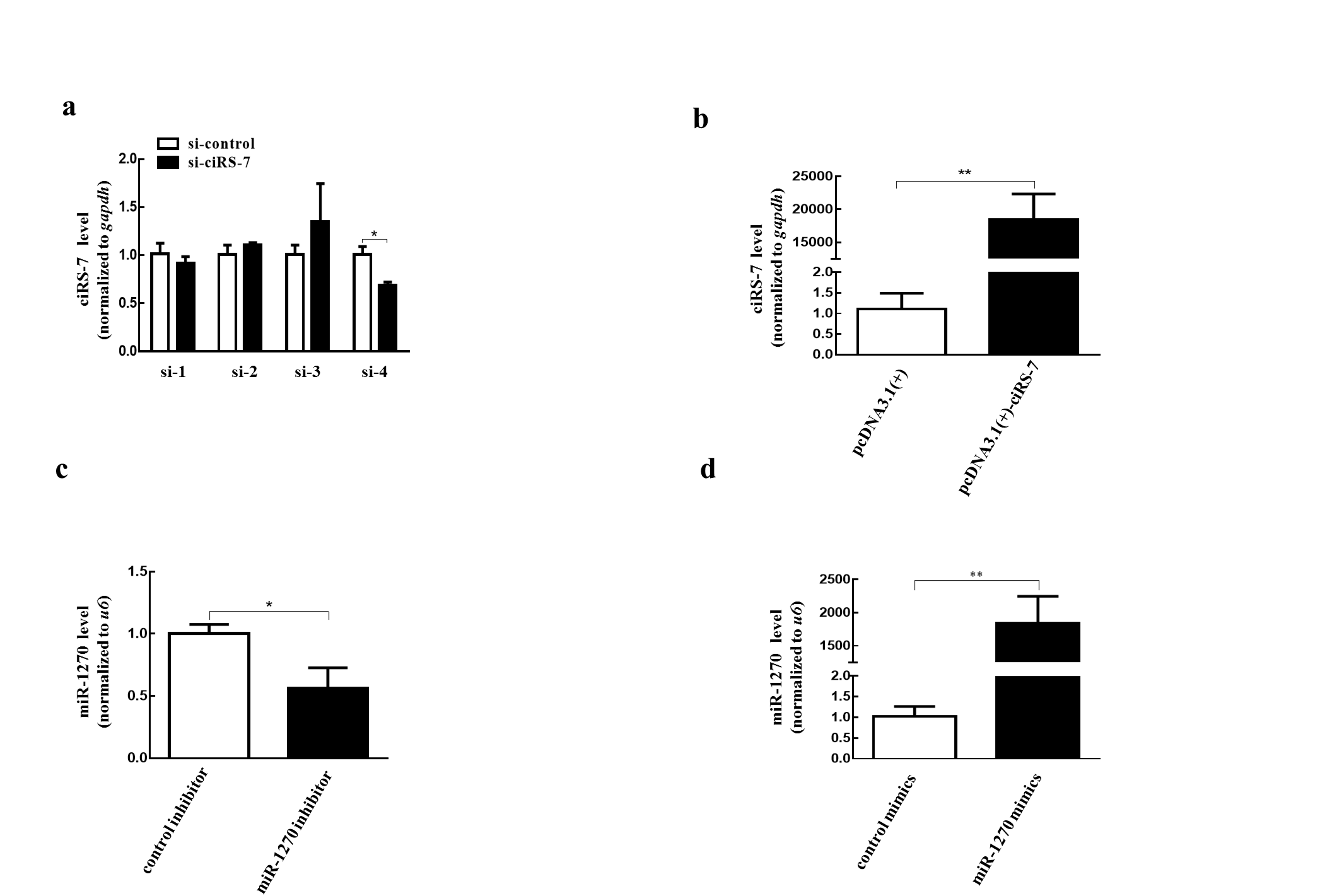

Supplement: Supplementary file 9 — Additional file 9: Figure S4. ciRS-7 and miR-1270 were successfully inhibited or overexpressed in HCT-8 cells. HCT-8 cells were transfected with four siRNAs targeting ciRS-7 (a), pcDNA3.1(+)-ciRS-7 plasmid (b), miR-1270 inhibitor (c), and miR-1270 mimics (d) for 24 h, and the expression levels of ciRS-7 or miR-1270 were analyzed by qRT-PCR. The data represent the mean ± SD of three independent experiments. *P < 0.05, **P < 0.01 (TIF 332 KB) [file 13071_2021_4739_MOESM9_ESM.tif]

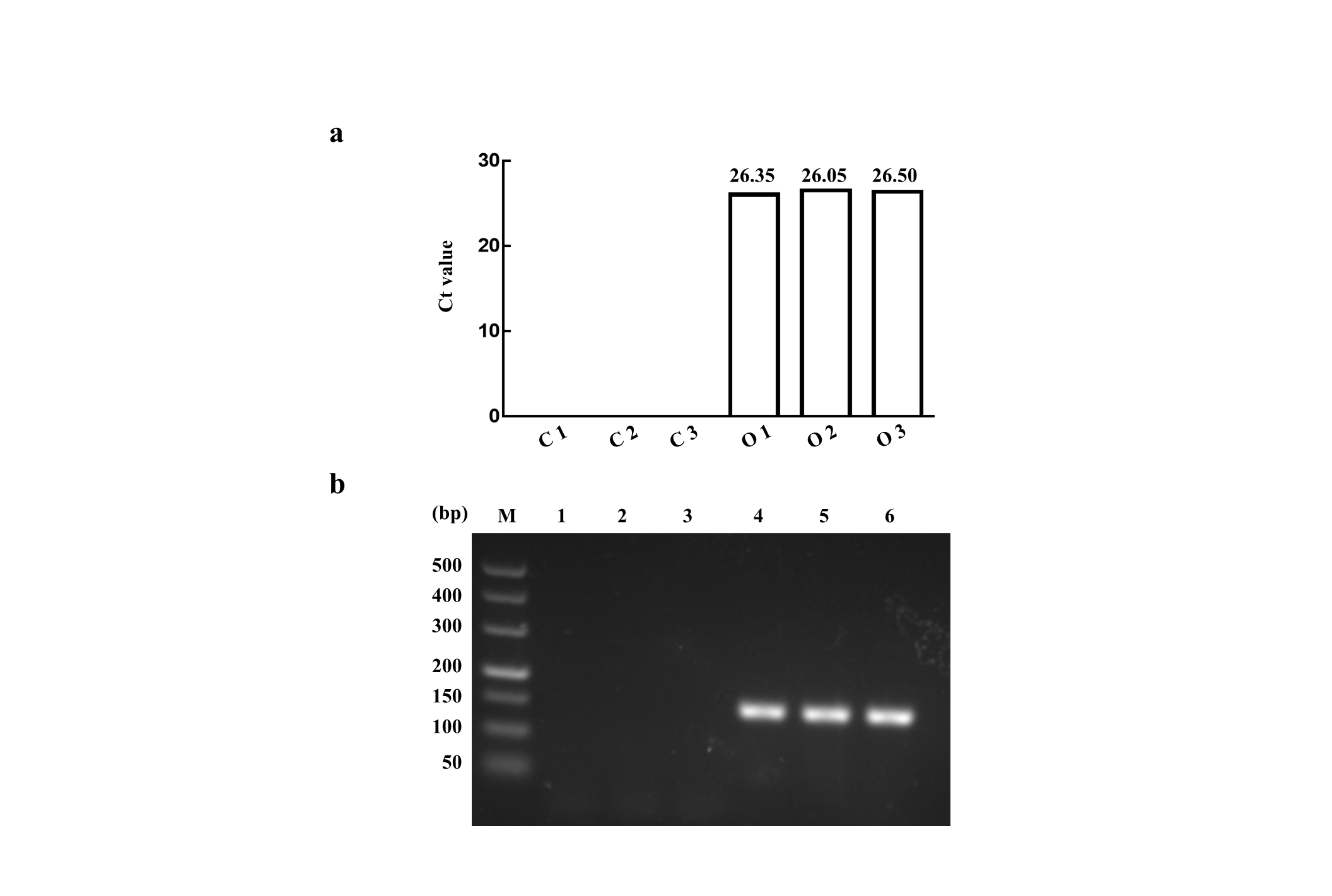

Supplement: Supplementary file 10 — Additional file 10: Figure S5. The expression of Crytposporidium hsp70 in HCT-8 cells infected with C. parvum. a. The Ct values of Crytposporidium hsp70 for each HCT-8 cell sample with (O1-O3) or without (C1-C3) C. parvum infections obtained from qRT-PCR assays. b. Agarose gels of qRT-PCR assays. Lane M represents DL500 DNA Marker (TIF 379 KB) [file 13071_2021_4739_MOESM10_ESM.tif]

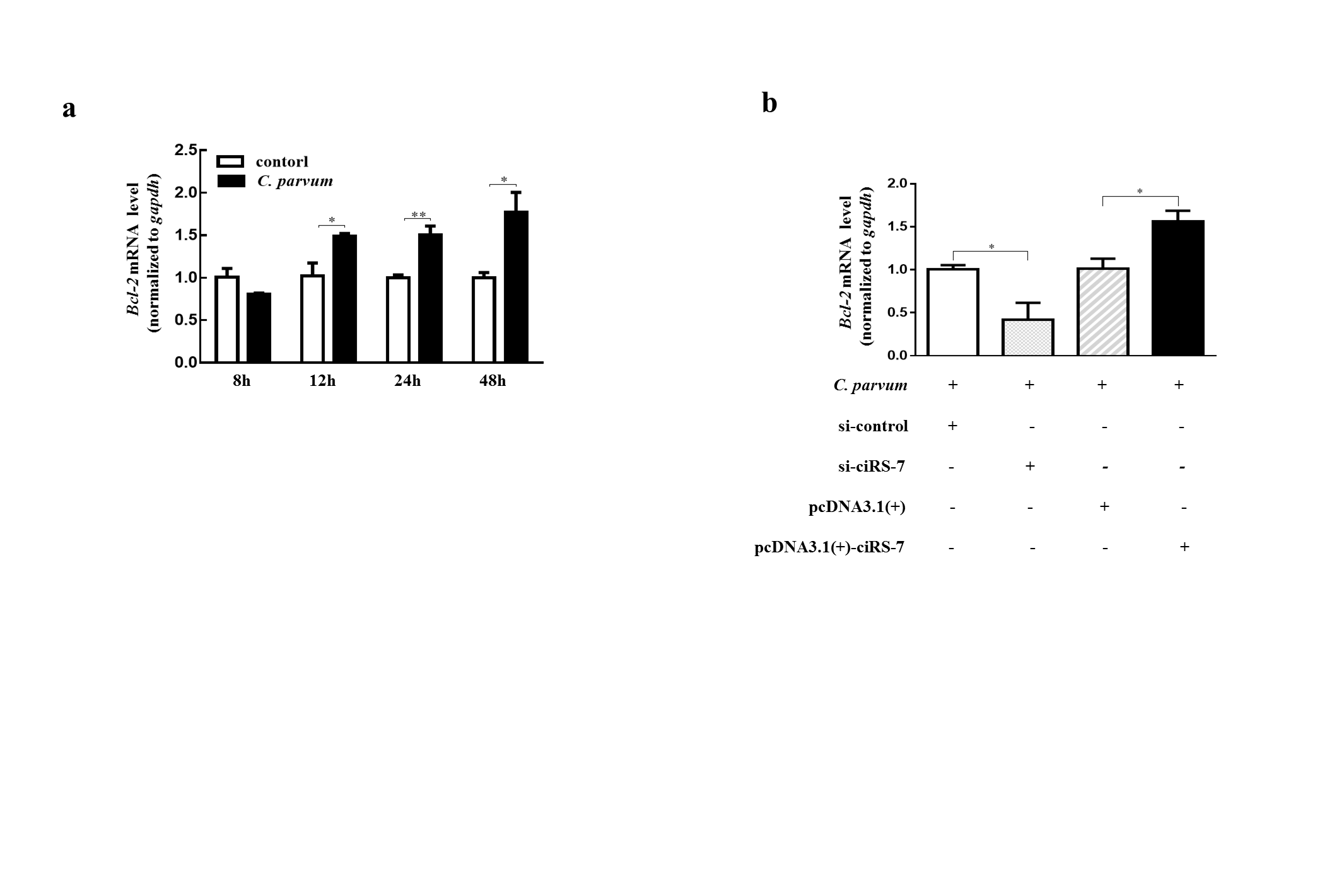

Supplement: Supplementary file 11 — Additional file 11: Figure S6. Bcl-2 expression in HCT-8 cells following C. parvum infection. a Time-dependent expression of Bcl-2 mRNA levels in HCT-8 cells during C. parvum infection. b Bcl-2 mRNA levels in HCT-8 cells transfected with pcDNA3.1(+)-ciRS-7 plasmid or si-ciRS-7 and exposed to C. parvum infection for 24 h. The data represent the mean ± SD for three independent experiments. *P < 0.05, **P < 0.01 (TIF 299 KB) [file 13071_2021_4739_MOESM11_ESM.tif]
